# An unexpected role of EasD_af_: catalyzing the conversion of chanoclavine aldehyde to chanoclavine acid

**DOI:** 10.1007/s00253-024-13157-8

**Published:** 2024-05-07

**Authors:** Zhi-Pu Yu, Chunyan An, Yongpeng Yao, Ju-Zhang Yan, Shu-Shan Gao, Yu-Cheng Gu, Chang-Yun Wang, Chengsen Cui

**Affiliations:** 1 Key Laboratory of Marine Drugs, The Ministry of Education of China, Institute of Evolution & Marine Biodiversity, School of Medicine and Pharmacy, Ocean University of China, Qingdao, 266003 People’s Republic of China; 2 Laboratory for Marine Drugs and Bioproducts, Qingdao National Laboratory for Marine Science and Technology, Qingdao, 266003 People’s Republic of China; 3 Beijing Institute for Drug Control, NMPA Key Laboratory for Research and Evaluation of Generic Drugs, Beijing Key Laboratory of Analysis and Evaluation on Chinese Medicine, Beijing, 102206 People’s Republic of China; 4 State Key Laboratory of Mycology, Institute of Microbiology, Chinese Academy of Sciences, Beijing, 100101 People’s Republic of China; 5 Tianjin Institute of Industrial Biotechnology, Chinese Academy of Sciences, Tianjin, 300308 People’s Republic of China; 6 Syngenta Jealott’s Hill International Research Centre, Bracknell, Berkshire, RG42 6EY UK

**Keywords:** Ergot alkaloid, EasD_af_, CC acid, Oxidations

## Abstract

**Abstract:**

Ergot alkaloids (EAs) are a diverse group of indole alkaloids known for their complex structures, significant pharmacological effects, and toxicity to plants. The biosynthesis of these compounds begins with chanoclavine-I aldehyde (CC aldehyde, **2**), an important intermediate produced by the enzyme EasD_af_ or its counterpart FgaDH from chanoclavine-I (CC, **1**). However, how CC aldehyde **2** is converted to chanoclavine-I acid (CC acid, **3**), first isolated from *Ipomoea violacea* several decades ago, is still unclear. In this study, we provide in vitro biochemical evidence showing that EasD_af_ not only converts CC **1** to CC aldehyde **2** but also directly transforms CC **1** into CC acid **3** through two sequential oxidations. Molecular docking and site-directed mutagenesis experiments confirmed the crucial role of two amino acids, Y166 and S153, within the active site, which suggests that Y166 acts as a general base for hydride transfer, while S153 facilitates proton transfer, thereby increasing the acidity of the reaction.

**Key points:**

***•*** *EAs possess complicated skeletons and are widely used in several clinical diseases*

***•*** *EasD*_*af*_ *belongs to the short-chain dehydrogenases/reductases (SDRs) and converted CC or CC aldehyde to CC acid*

***•*** *The catalytic mechanism of EasD*_*af*_ *for dehydrogenation was analyzed by molecular docking and site mutations*

**Supplementary Information:**

The online version contains supplementary material available at 10.1007/s00253-024-13157-8.

## Introduction

**Fig. 1:**
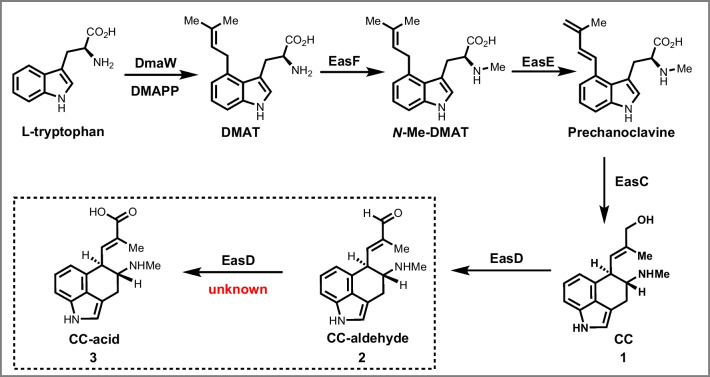
The biosynthetic pathway of CC acid. The procedure from precursor L-tryptophan and DMAPP to intermediate CC aldehyde has been clearly elucidated in several literatures. The catalytic role of EasD_af_ for the formation of CC acid has never been reported (marked by dotted line)

Ergot alkaloids were initially isolated from grass and grain infected with *Claviceps* fungi (Wallwey and Li 2011) and later found in other filamentous fungi and plants due to their importance in treating neurological diseases like migraines, uterine hemorrhage, and Parkinson’s disease (Fabian et al. 2018; Wallwey and Li 2011). In 2019, Gao’s group made a groundbreaking discovery that the catalase EasC catalyzes the conversion of prechanoclavine (PCC) to CC (**1**) (Fig. 1) (Yao et al. 2019). This led to the complete understanding of the biosynthetic pathway for the common intermediate CC aldehyde (**2**) derived from tryptophan (Fig. 1). The pathway includes the actions of various enzymes, including DmaW, EasF, EasE, and EasC, producing N-methyl-dimethylallyltryptophan (N-DMAT), followed by PCC, and finally, CC and CC aldehyde (Fig. 1) (Chen et al. 2017; Wallwey et al. 2010).

Protein EasD_af_ has been documented as the catalyst responsible for oxidizing CC **1** in the early biosynthetic pathway of EA, relying on NAD^+^ to convert CC **1** to CC aldehyde **2** (Wallwey et al. 2010). EasD_af_ belongs to the short-chain dehydrogenase/reductase (SDRs) family, one of the most abundant subfamilies of alcohol dehydrogenases (Zhou et al. 2020). Although it shares little sequence identity with classical SDRs, it contains two characteristic motifs, including a YXXXK sequence and a TGXXX[AG]XG sequence corresponding to the catalytic site featuring a conserved tyrosine and the dinucleotide-binding site, respectively (Figure S1).

The chemical structure of CC acid (CC acid, **3**) was initially identified in the seeds of *Ipomoea violacea* (Choong and Shough 1977), and the first total synthesis of CC acid **3** was achieved chemically using 3-indolecarboxaldehyde (Somei et al. 1990). However, no reports exist on the transformation of CC aldehyde **2** to CC acid **3** in literature. Considering aldehydes can pose risks of toxicity, mutagenicity, and carcinogenicity, enzymes that convert aldehyde to acid can be beneficial for the producing host (Fan et al. 2006). In our study, we demonstrate for the first time that EasD_af_ possesses the ability to catalyze the conversion of CC **1** or CC aldehyde **2** to CC acid **3** under in vitro conditions.

## Material and methods

### General materials and methods

The authentic compound CC (Figure S3) was isolated from the recombinant strain that has been modified to overexpress the biosynthetic gene cluster of CC in *Aspergillus nidulans* and overproduce CC in our laboratory (Yao et al. 2022). Another authentic compound CC aldehyde (Figure S4) was synthesized as a published reference (Chaudhuri et al. 2018). The strain of overproducing CC contains three plasmids that equal to two copies of *easF*, two copies of *dmaW*, three copies of *easE*, and two copies of *easC* (Table S1). The analytical grade of chemicals and solvents was purchased and used in this study. All buffers and solutions were made by Milli-Q water. Fast mutagenesis system kits and BL21(DE3) chemically competent cells were purchased from TransGen Biotech (Beijing, China). Tsingke Biotechnologies (Beijing, China) performed DNA sequencing and primers synthesizing. High-performance liquid chromatography (HPLC) analysis was performed on a CORUI (China) system using a C_18_ analytical column (Agilent Eclipse Plus- C_18_, 4.6 × 250 mm, 5.0 μm). LCMS analysis was performed on the Waters-2695HPLC/2996MS (USA) system using C_18_ analytical column (Agilent Eclipse XDB- C_18_, 2.1 × 100 mm, 3.5 μm). A semi-preparative HPLC (Waters 2695 system, USA) was used to complete the isolation and purification through a C_18_ semi-preparative Welch column (250 × 10 mm, 5 μm, 25 °C, flow 2.0 mL/min). Acetonitrile and methanol in our study were HPLC grade and purchased from Sigma-Aldrich (St. Louis, MO, USA).

### Site-mutation and protein purification

The plasmid pET28a of EasD_af_ (Sequence ID: Q4WZ66.1) from *A. fumigatus* (CGMGCC 3.772) was constructed using *Nde*I/*Xho*I as inserting sites by Gibson method, stored in our laboratory, and transferred into the competent cells of *E. coli* BL21(DE3) (Yu et al. 2022). Site-mutation experiment was performed on reverse PCR amplification by partially overlapping and site-mutated primers (Table S2) and template of wild-type methylated plasmid using fast mutagenesis system kit. The resultant plasmids were digested by DMT restriction enzymes in vitro and then transformed into the competent cells of *E. coli* BL21(DE3) and plated on LB solid medium with 50 mg/mL kanamycin at 37 °C overnight. The correct transformant was cultivated in a 10 mL LB liquid medium of 50 mg/mL kanamycin at 37 °C and 220 rpm overnight, which was then scaled up into 2-L LB liquid medium to express corresponding proteins induced by isopropyl-*β*-D-thiogalactoside (IPTG, 0.4 mM). The bacteria were collected and suspended in Tris–HCl buffer and then sonicated by an ultrasonic processor (SX-605D, Henglong Instrument Co. Ltd., Changzhou, China). A Thermo Scientific Sorvall ST 16R centrifuge (Thermo Scientific, Germany) was used to centrifuge the bacterial lysate, and nickel resin in a 5 mL HisTrap™HP (GE Healthcare Life Science, Uppsala, Sweden) was mixed with obtained supernatant and purified proteins. The purification procedures have been as follows: the above mixture was subjected into a column and washed using 3 column volume of buffer B (Tris–HCl 20 mM, NaCl 300 mM, imidazole 40 mM, and glycerin 10%). Then, the bonded protein was eluted using buffer C (Tris–HCl 20 mM, NaCl 300 mM, imidazole 250 mM, and glycerin 10%) and concentrated using exchange buffer by 10 kDa Amicon Ultra (Merck Millipore). Finally, SDS-PAGE (Figure S2) and Bradford assay were respectively used to analyze the protein purity and determine the protein concentration using bovine serum albumin (BSA) as a standard.

### Enzyme assays

Biochemical assays with the substrate CC or CC aldehyde were both performed in a 50 μL system, containing 2 mM substrate, 0.5 mM enzymes, 20 mM NAD^+^, and 50 mM phosphate buffer solution (pH 7.0) in a 2 mL Eppendorf tube. This reaction mixture was incubated at 30 °C for 3 or 5 h and quenched with 100 μL methanol, which was then thoroughly mixed and centrifuged at 12,000 g for 5 min. A 0.22-μm microfilter was used to filter the obtained supernatant, which was subjected to HPLC and LC-MS for further analyses. The enzyme activity of the wild-type EasD_af_ was set as the reference at 100%. The activities of site-mutated proteins were then determined and calculated based on the relative conversion rates of substrates.

### HPLC and LC-MS analyses

HPLC analysis was performed on Clarity of CORUI using C_18_ analytical column (Agilent Eclipse Plus-C_18_, 4.6 × 250 mm, 5.0 μm) and diode array detector. All samples were integrated at 280 nm. This elution gradient carried out three steps of 5−55% acetonitrile (MeCN)-H_2_O (v/v, 0.1% formic acid) in 15 min and followed by 55−95% acetonitrile (MeCN)-H_2_O (v/v, 0.1% formic acid) in 5 min and 95% acetonitrile (v/v, 0.1% formic acid) for 3 min with a flow rate of 1 mL/min. The injection volume was 20 μL for all samples.

LC-MS analysis was performed on Waters 2596 equipped with a mass spectrum detector (2996) using C_18_ column (Agilent Eclipse XDB- C_18_, 2.1 × 100 mm, 3.5 μm). The HPLC analysis was eluted by gradient method (0.5 mL/min, 20 min, H_2_O/MeCN, 90/10→0/100, v/v) and detected by 280 nm and molecule weight 271 of positive ion mode.

### Isolation and purification of CC acid by semi-preparative HPLC

To obtain the catalytic product, the enzyme assay of EasD_af_ and CC was scaled up to 20 mL. After incubation, the pH of the reaction mixture was initially adjusted to the PI value (5.0) of EasD_af_, resulting in the precipitation of most of the protein. After complete protein precipitation, high-speed centrifuge was performed for 10 min, followed by pH adjustment to 7.0, and the supernatant was concentrated using a rotary evaporator. The resulting mixture was dissolved in chromatographic-grade methanol, filtered in 0.45 μm microfilter, and subjected to semi-prepared HPLC chromatography using a Welch column (250 × 10 mm, 5 μm, 25 °C, flow 2 mL/min) with an isocratic wash of 20% MeCN in water with 0.1% formic acid. The solvent and water were removed, and the purified product was concentrated under reduced pressure. The compound was dissolved in chromatographic-grade MeOH and confirmed using LC-MS. The structure of CC acid was elucidated by analyzing its 2D NMR spectra and comparing it with that of CC.

### Molecular docking analysis

The EasD_af_ structure was obtained from the AlphaFold protein structure database (https://alphafold.ebi.ac.uk/). The preprocessing of substrates, EasD_af_, and substrate-enzyme interaction was performed by AutoDock Tools and Vina V_1.5_ and exhibited using PyMOL V_2.5_ (The PyMOL Molecular Graphics System: version 2.5 Schrodinger, LLC, https://pymol.org/2/).

## Results

### In vitro biochemical reaction of EasD_af_ with CC

**Fig. 2:**
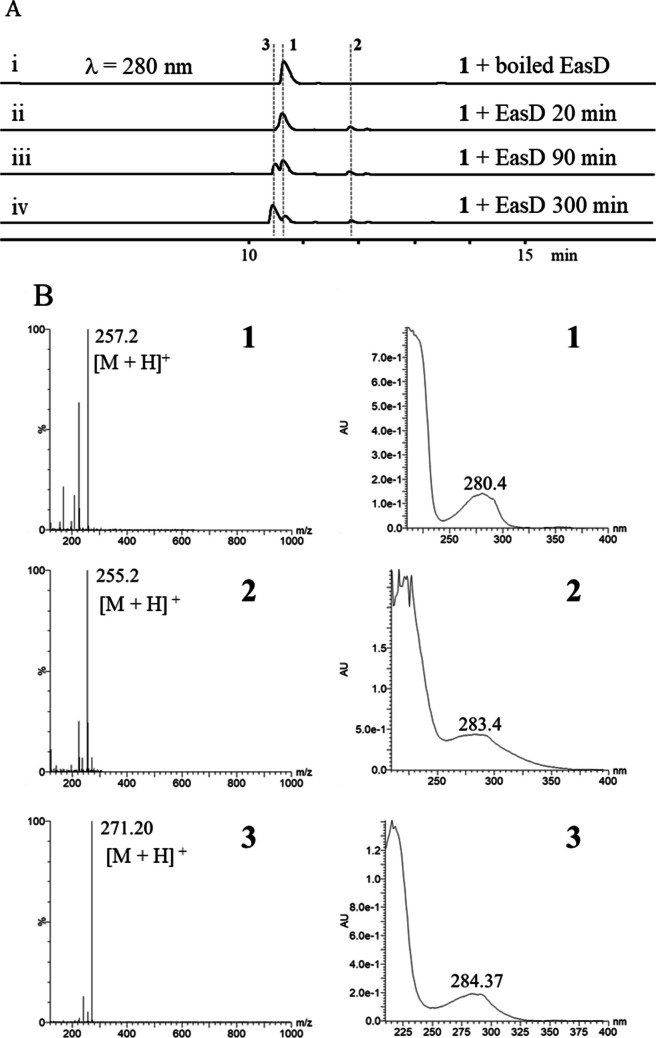
Analyses of products of reactions catalyzed by EasD_af_. **A** HPLC trace of EasD_af_ time-course reaction during 300 min. The retention time of substrate **1** and productions **2** and **3** is at 10.6, 11.9, and 10.5 min, respectively. **B** Mass spectrometry data (left) and UV spectra (right) of three compounds

The coding region of the EasD_af_ gene from *Aspergillus fumigatus* (CGMGCC 3.772) was synthesized and inserted into the pET28a plasmid (Yu et al. 2022). EasD_af_ protein was further purified from *Escherichia coli* BL21 (DE3). Biochemical reactions were performed using a 50 μL system consisting of 2 mM substrate, 0.5 mM enzyme, and 20 mM NAD^+^ in phosphate buffer at pH 7.0. After incubating at 30 °C for 20, 90, and 300 min, respectively, the reaction mixtures were quenched using 100 μL of chromatography-grade methanol. Analysis via HPLC toward the reaction mixture revealed two new peaks **2** and **3**, which is 1.3 min later and 0.1 min earlier than **1** in the HPLC trace, respectively (Fig. 2A). Both **2** and **3** share the same ultraviolet (UV) absorption spectrum as **1** (Fig. 2B). Mass spectral data (Fig. 2B) suggested the molecular weight (MW) of **2** and **3** is 254 and 270, respectively, which corresponds to 2 unit less and 14 unit more than that of **1** (MW = 256), respectively (Fig. 2B).

### Assigning the chemical structure of product 3 as CC acid

Compound **2** was identified as CC aldehyde and verified by comparison with a synthetic sample in our lab (Figure S4). Although **3** was previously isolated and characterized from *Ipomoea violacea* (Choong and Shough 1977), the catalytic function of EasD_af_ in its biosynthesis had not been reported. To confirm this newly observed product, a large-scale EasD_af_-reaction was performed, and the mixture was purified and identified using HPLC and analyzed with LC-MS (Fig. 2B) and 2D NMR (Figure S5 ~ S9).

**Fig. 3:**
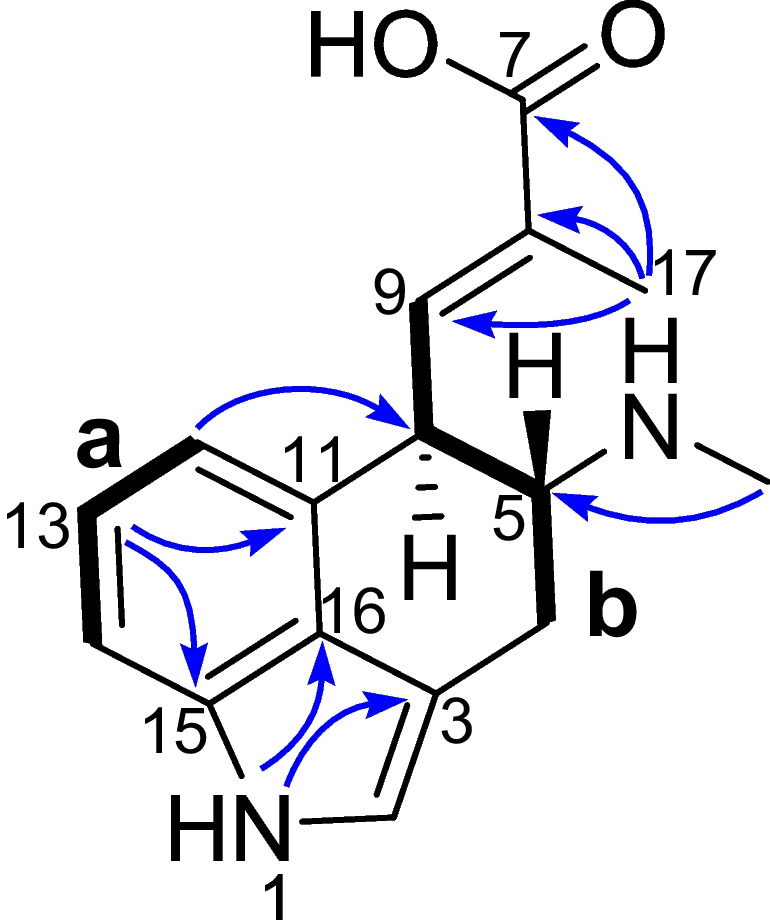
Key ^1^H-^1^H COSY (  ) and HMBC (  ) correlations for **3**

Product **3** displayed a protonated molecular ion peak at *m/z* 271.2 in the MS experiment, consistent with a molecular formula of C_16_H_18_N_2_O_2_ and nine indices of hydrogen deficiency. Comparing the NMR data of compound **3** with that of compounds **1** and **2** in the literature (Yao et al. 2019) revealed that they were structural analogs, with the difference occurring to the functional group at C-7 (Fig. 3). The signals for a carboxy group (*δ*_H_ ND, *δ*_C_ 169.9) in **3** were in place of those for aldehyde group (*δ*_H_ 9.51, *δ*_C_ 196.0) in **2** and carbinol group (*δ*_H_ 3.93, 3.93, *δ*_C_ 66.7) in **1** (Yao et al. 2019). Analysis of the ^1^H and ^13^C NMR data (Table 1) revealed the characteristic resonances for an indole moiety (*δ*_H_ 10.70, 7.15, 6.98, 6.50; *δ*_C_ 133.8, 129.3, 125.7, 122.2, 119.5, 115.1, 109.6, 108.9), conjugated carboxyl group (*δ*_C_ 169.9), a trisubstituted double bond (*δ*_H_ 6.55, *δ*_C_ 139.4, 131.9), a *N*-methyl group (*δ*_H_ 2.37, *δ*_C_ 32.6), and a single methyl group (*δ*_H_ 1.94, *δ*_C_ 13.3). The indole moiety, carboxyl group, and double bond accounted for eight indices of hydrogen deficiency, the remaining one being attributable to tricyclic architecture for **3**. Inspection of ^1^H–^1^H COSY data revealed two spin-spin coupling systems of **a** (H-12 to H-14) and **b** (H_2_-4 with H-5, H-5 with H-10, and H-9 with H-10) as drowned in bold bonds (Fig. 3). These two fragments were linked together via aromatic carbon (C-11, *δ*_C_ 129.3) by the HMBC correlations (Fig. 3) from H-12 to C-10 (*δ*_C_ 42.6), indicating a tricyclic EA skeleton. The carboxyl group was connected via C-8 (*δ*_C_ 131.9) to C-9 (*δ*_C_ 139.4) based on the HMBC correlations from H_3_-17 to C-7 (*δ*_C_ 169.9), C-8, and C-9. Additionally, the HMBC correlated data between *N*-CH_3_ (*δ*_H_ 2.37) and C-5 (*δ*_C_ 60.4) confirmed the substitution position of this methyl group. Furthermore, the relative and absolute configurations of stereocenters were assigned to be the same as their counterparts of **1** and **2** by comparing NMR data. In short, the structure of product **3** was elucidated to be same as that of CC acid. Therefore, we have confirmed enzyme EasD_af_ could unexpectedly catalyze the formation of CC acid from CC or CC aldehyde, in addition to its original activity of producing CC aldehyde in the early synthetic pathway of EAs.

**Table 1:** ^1^H NMR (500 MHz) and ^13^C NMR (125 MHz) data of CC acid in DMSO-*d*_6_

No.	*δ*_H_, multi. (*J* in Hz)	*δ*_C_, multi. (*J* in Hz)
1-NH	10.70, s	
2	7.01, s	119.5
3		108.9
4	2.66, m 3.13, m	ND
5	2.88, m	60.4
6-NH	ND	
7-COOH	ND	169.9
8		131.9
9	6.55 d (10.2)	139.4
10	3.93, dd (10.9, 7.0)	42.6
11		129.3
12	6.50, d (7.1)	115.1
13	6.98, dd (8.1, 7.1)	122.2
14	7.15, d (8.1)	109.6
15		133.8
16		125.7
17-CH_3_	1.94, s	13.3
6-CH_3_	2.37, s	32.6

### Proposed mechanism of EasD_af_ catalyzing the oxidation of CC or CC aldehyde

We discovered that EasD_af_ can function as both an alcohol and aldehyde dehydrogenase, catalyzing the conversion of CC to CC acid in addition to its already established alcohol oxidation function. This dual role is unique and differentiates it from other enzymes that typically perform either alcohol oxidation or aldehyde conversion. Only few enzymes exhibit similar functions, including choline oxidase, horse liver alcohol dehydrogenase (Fan et al. 2006), molybdoenzyme aldehyde oxidase, and 5-(hydroxymethyl)furfural oxidase (Troiano et al. 2020). Our finding demonstrates the versatility of EasD_af_ and expands the knowledge about its capabilities beyond what was previously known.

**Fig. 4:**
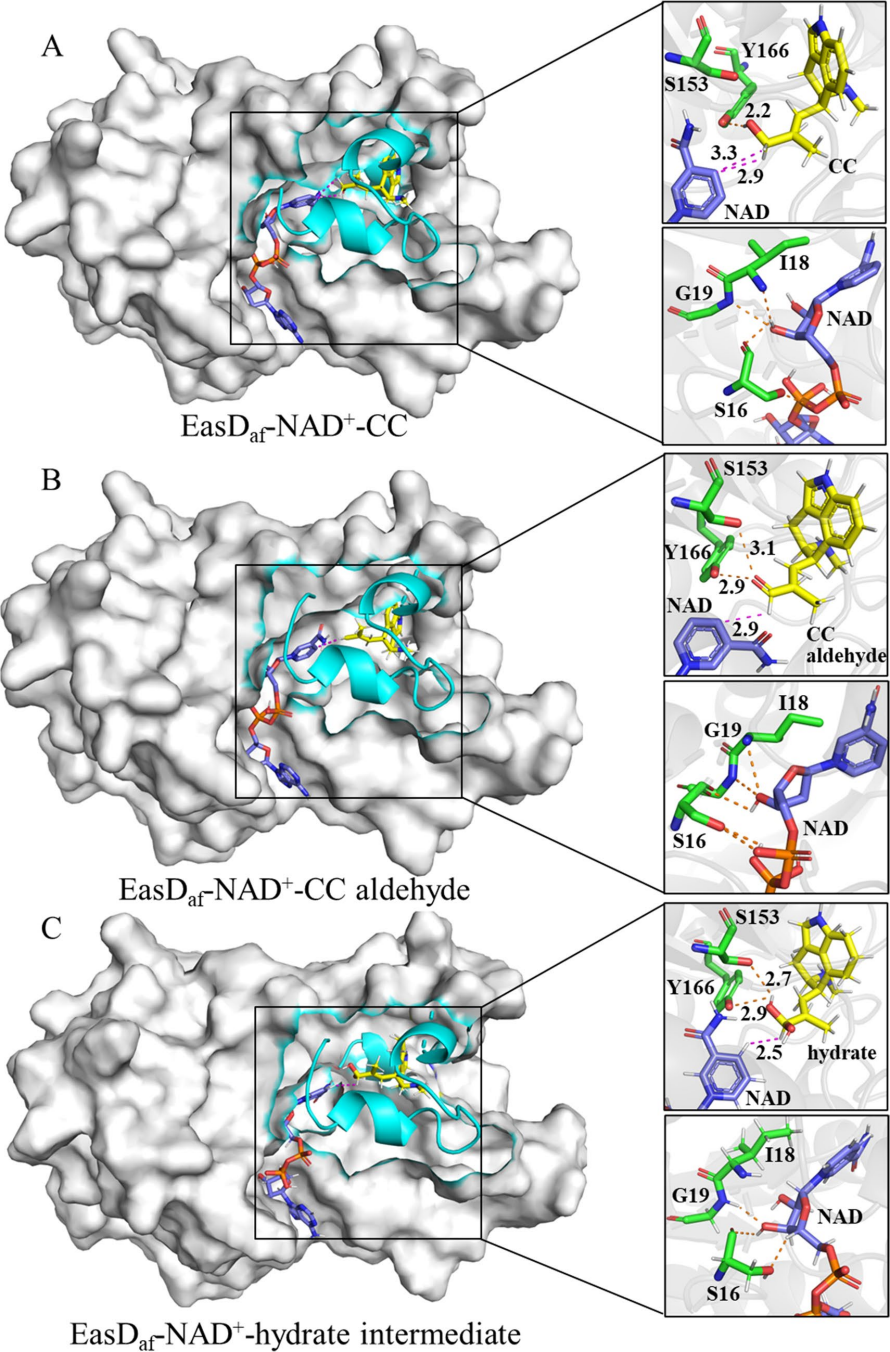
Molecular docking of 3D-structure of EasD_af_ with the substrates **1**, **2** and its *gem-diol* form intermediate. **A** Molecular docking results of EasD_af_ with **1** (CC). **B** Molecular docking results of EasD_af_ with **2** (CC aldehyde). **C** Molecular docking results of EasD_af_ with *gem-diol* form intermediate of **2**

We used AlphaFold protein structure database to predict the 3D structure of EasD_af_, which provided insight into the binding patterns between the enzyme, substrates, and cofactor during the catalytic process. The molecular docking simulation results showed strong interactions between NAD^+^ and conserved residues such as S16, I18, and G19 (Fig. 4), which belong to the typical cofactor-binding motif found in the sequence of short-chain dehydrogenase and reductase (Wallwey et al. 2010).

Molecular docking simulations demonstrated similar orientation of **1**, **2** and its *gem*-diol form intermediate within the active pocket, with the side chain derived from isopentenyl group positioned inside and the tricyclic moiety facing outward. The hydrogens of the carbinol group at C-7 in **1** and the aldehyde group at C-7 in **2** and its *gem*-diol form intermediate pointed toward the C4 of NAD^+^, showing proximity of approximately 2.5 to 3.3 Angstroms (Fig. 4). This arrangement suggests efficient hydrogen transfer between substrates and the cofactor during catalysis.

**Fig. 5:**
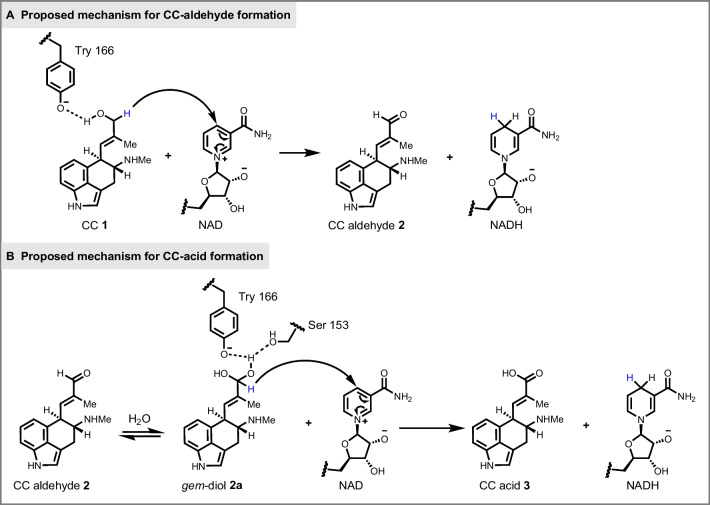
Proposed mechanism of EasD_af_ catalyzing substrates **1**-**2** to corresponding oxidation products. **A** Proposed mechanism of EasD_af_ converting **1** (CC) to **2** (CC aldehyde). **B** Proposed mechanism of EasD_af_ converting **2** (CC aldehyde) to **3** (CC acid)

Our studies of the molecular mechanism of EasD_af_’s dehydrogenation process involved in the conversion of CC to CC aldehyde were based on previous research on alcohol and aldehyde dehydrogenases (Fan et al. 2006; Riegert et al. 2017). As outlined in Fig. 5A, according to molecular docking simulations, it appears that EasD_af_ follows a similar process used by short-chain dehydrogenases and reductases (Riegert et al. 2017), specifically the abstracting of a hydrogen atom from the C-7 hydroxyl group of **1** by a conserved tyrosine residue in the tyrosinate form, which functions as a general base, allowing for hydride transfer from C-7 of CC to the C-4 of the bound NAD^+^.

On the other hand, there is limited literature on the catalytic mechanism of aldehyde dehydrogenase. Previous research (Fan et al. 2006) suggested that a *gem*-diol form intermediate **2a** (Fig. 5B) must be formed prior to dehydrogenation by the addition of a water molecule to CC aldehyde, which is a necessary step in the conversion of CC aldehyde to CC acid. According to molecular docking simulations, two conserved residues, instead of only one essential residue Y166 in the conversion of CC **1** to CC aldehyde **2**, Y166 and S153 are essential to this reaction. Y166 is proposed to act as a general base facilitating hydride transfer from **2** to NAD^+^, which is similar to its role in the oxidation of CC. S153 is proposed to stabilize the *gem*-diol intermediate **2a** and increase the acidity of the hydroxy group (Krishnakumar et al. 2006), assisting in proton transfer from the active site to the external environment (Olson et al. 1996). In conclusion, we proposed that both Y166 and S153 play vital roles in the enzymatic process of EasD_af_. Y166 is critical to both steps of dehydrogenations, while S153 is proposed to be involved in facilitating the conversion of CC aldehyde to CC acid.

### Mutation results of Y166 and S153

Based on the molecular docking results, we focused on the distinct roles of two residues in the oxidation process: Y166 interacts with both **1** and **2**, while S153 only interacts with **2**. To validate the different roles of Y166 and S153 in the catalytic mechanism of EasD_af_, we conducted site-directed mutagenesis experiments using alanine scanning, creating two mutant strains Y166A and S153A.

**Fig. 6:**
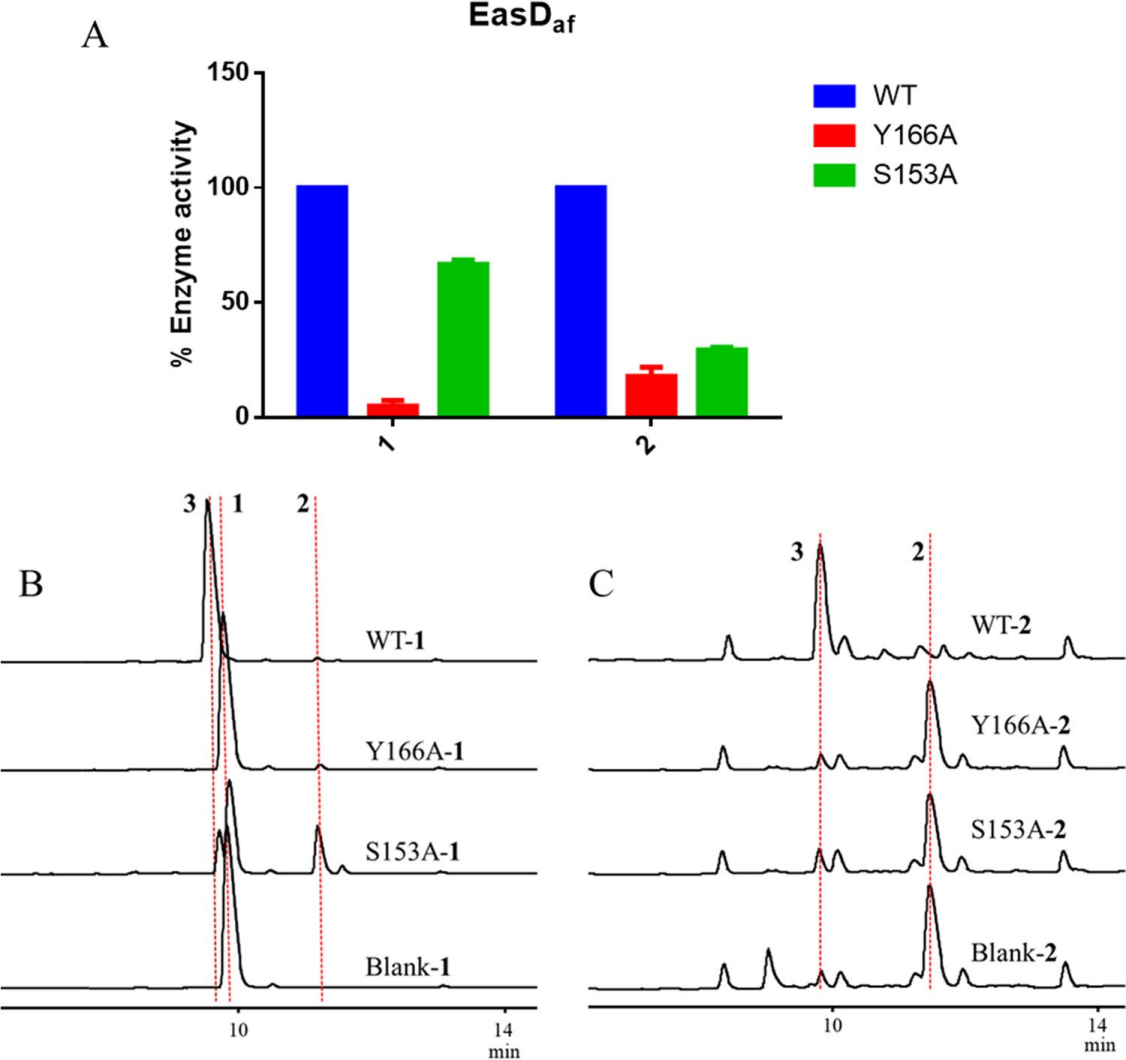
Comparison of the dehydrogenation activities between wild type and mutants of EasD_af_. **A** The relative enzyme activities of Y166A and S153A for **1** and **2**. **B** HPLC analysis of biochemical reaction in vitro using purified protein for **1**. **C** HPLC analysis of biochemical reaction in vitro using purified protein for **2**

As depicted in Fig. 6, the purified mutant strain Y166A displayed reduced activity compared to the wild-type enzyme across all conditions, indicating the essential role of Y166 in the whole catalysis. Conversely, S153A demonstrated variable activity levels dependent on the substrate: retaining 66.6% efficiency when oxidizing **1** but decreasing to only 29.3% when acting upon **2** (Fig. 6A), highlighting its primary contribution to the second stage of the reaction pathway. Overall, Y166 appears to be integral throughout the entire conversion process, while S153 has a greater impact on the later steps of the transformation.

## Discussion

Enzyme EasD_af_, identified as an NAD^+^-dependent dehydrogenase, has previously been associated with producing CC aldehyde **2** in EA biosynthesis (Jakubczyk et al. 2014). However, references lacked evidence of its ability to oxidize the substrate aldehyde **2**. Our research has uncovered new knowledge of its function by establishing an in vitro biochemically relevant reaction system and performing molecular docking and site mutation analysis. We confirmed that EasD_af_ directly converts both CC **1** and CC aldehyde **2** to produce CC acid **3**, a well-known compound isolated from *Ipomoea violacea* (Choong and Shough 1977). This discovery establishes EasD_af_’s dual functionality, enabling the transformation of both CC and CC aldehyde into CC acid in vitro.

According to literature (Wallwey et al. 2010), EasD_af_ consists of 261 amino acids with a molecular mass of 27.8 kDa and features two conserved motifs of typical short-chain dehydrogenases reductases (Figure S1 ~ S2), which was consistent with our molecular docking and site-mutation results. Our experimentation validates the vital role of conserved residue Y166 in two steps of dehydrogenation and S153 in forming CC acid. The significance of this study lies in the potential dangers posed by unconverted aldehydes (Fan et al. 2006), such as toxicological hazards, mutagenicity, and carcinogenicity, which motivates the development of methods for efficiently converting them to safer intermediates. EasD_af_ has the ability to catalyze the conversion of chanoclavine-I aldehyde into chanoclavine-I acid, a non-toxic compound to organisms. Therefore, EasD_af_ represents a valuable contribution to the exploration, especially considering the limited existing research in this field.

## Supplementary information

ESM 1 (PDF 1165 kb)

## Data Availability

Data and material described in this study are available from the authors upon reasonable request and availability.

## References

[CR1] Chaudhuri S, Bhunia S, Roy A, Das MK, Bisai A (2018) Biomimetic total syntheses of clavine alkaloids. Organic Letters 20(1):288–291. 10.1021/acs.orglett.7b03683 29235356 10.1021/acs.orglett.7b03683

[CR2] Chen JJ, Han MY, Gong T, Yang JL, Zhu P (2017) Recent progress in ergot alkaloid research. RSC Adv 7(44):27384–27396. 10.1039/c7ra03152a

[CR3] Choong TC, Shough HR (1977) The isolation and synthesis of chanoclavine-I acid. Tetrahedron Lett 36:3137–3138

[CR4] Fabian SJ, Maust MD, Panaccione DG (2018) Ergot alkaloid synthesis capacity of *Penicillium camemberti*. Appl Environ Microbiol 84(19):e01583–e01518. 10.1128/aem.01583-18 30076193 10.1128/AEM.01583-18 PMC6146994

[CR5] Fan F, Germann MW, Gadda G (2006) Mechanistic studies of choline oxidase with betaine aldehyde and its isosteric analogue 3,3-dimethylbutyraldehyde. Biochemistry 45(6):1979–1986. 10.1021/bi0517537 16460045 10.1021/bi0517537

[CR6] Jakubczyk D, Cheng JZ, O'Connor SE (2014) Biosynthesis of the ergot alkaloids. Nat Prod Rep 31(10):1328–1338. 10.1039/c4np00062e 25164781 10.1039/c4np00062e

[CR7] Krishnakumar AM, Nocek BP, Clark DD, Ensign SA, Peters JW (2006) Structural basis for stereoselectivity in the (R)- and (S)-hydroxypropylthioethanesulfonate dehydrogenases. Biochemistry 45(29):8831–8840. 10.1021/bi0603569 16846226 10.1021/bi0603569

[CR8] Olson LP, Luo J, Almarsson O, Bruice TC (1996) Mechanism of aldehyde oxidation catalyzed by horse liver alcohol dehydrogenase. Biochemistry 35(30):9782–9791. 10.1021/BI952020X 8703951 10.1021/bi952020x

[CR9] Riegert AS, Thoden JB, Schoenhofen IC, Watson DC, Young NM, Tipton PA, Holden HM (2017) Structural and biochemical investigation of PglF from *Campylobacter jejuni* reveals a new mechanism for a member of the short chain dehydrogenase/reductase superfamily. Biochemistry 56(45):6030–6040. 10.1021/acs.biochem.7b00910 29053280 10.1021/acs.biochem.7b00910 PMC6211297

[CR10] Somei M, Mukaiyama H, Nomura Y, Nakagawa K (1990) The first total synthesis of (±)-chanoclavine-I acid and an alternative total synthesis of (±)-chanoclavine-I. Heterocycles 31(11):1919–1921. 10.3987/COM-90-5577

[CR11] Troiano D, Orsat V, Dumont MJ (2020) Status of biocatalysis in the production of 2,5-furandicarboxylic acid. ACS Catal 10(16):9145–9169. 10.1021/acscatal.0c02378

[CR12] Wallwey C, Li SM (2011) Ergot alkaloids: structure diversity, biosynthetic gene clusters and functional proof of biosynthetic genes. Nat Prod Rep 28(3):496–510. 10.1039/c0np00060d 21186384 10.1039/c0np00060d

[CR13] Wallwey C, Matuschek M, Li SM (2010) Ergot alkaloid biosynthesis in *Aspergillus fumigatus*: conversion of chanoclavine-I to chanoclavine-I aldehyde catalyzed by a short-chain alcohol dehydrogenase FgaDH. Arch Microbiol 192(2):127–134. 10.1007/s00203-009-0536-1 20039019 10.1007/s00203-009-0536-1

[CR14] Yao Y, An C, Evans D, Liu W, Wang W, Wei G, Ding N, Houk KN, Gao SS (2019) Catalase involved in oxidative cyclization of the tetracyclic ergoline of fungal ergot alkaloids. J Am Chem Soc 141(44):17517–17521. 10.1021/jacs.9b10217 31621316 10.1021/jacs.9b10217 PMC7592905

[CR15] Yao Y, Wang W, Shi W, Yan R, Zhang J, Wei G, Liu L, Che Y, An C, Gao SS (2022) Overproduction of medicinal ergot alkaloids based on a fungal platform. Metab Eng 69:198–208. 10.1016/j.ymben.2021.12.002 34902590 10.1016/j.ymben.2021.12.002

[CR16] Yu ZP, An C, Yao Y, Wang CY, Sun Z, Cui C, Liu L, Gao SS (2022) A combined strategy for the overproduction of complex ergot alkaloid agroclavine. Synth Syst Biotechnol 7(4):1126–1132. 10.1016/j.synbio.2022.08.003 36092273 10.1016/j.synbio.2022.08.003 PMC9428804

[CR17] Zhou J, Xu G, Ni Y (2020) Stereochemistry in asymmetric reduction of bulky-bulky ketones by alcohol dehydrogenases. ACS Catal 10(19):10954–10966. 10.1021/acscatal.0c02646

