# Supplementary material for: An unexpected role of EasDaf: catalyzing the conversion of chanoclavine aldehyde to chanoclavine acid
Source: Appl Microbiol Biotechnol. 2024 May 7;108(1):323. doi: 10.1007/s00253-024-13157-8 (PMC11076337; doi:10.1007/s00253-024-13157-8)
Supplement: Supplementary file 1 — (PDF 1165 kb) [file 253_2024_13157_MOESM1_ESM.pdf]

Supplementary Material

Journal: Applied Microbiology and Biotechnology

An Unexpected Role of EasD: Catalyzing the Conversion of Chanoclavine I Aldehyde to Chanoclavine I Acid

Zhi-Pu Yu[a,c], Chunyan An[e], Yongpeng Yao[d], Ju-Zhang Yan[b], Shu-Shan Gao[b], Yu-Cheng Gu[f]*, Chang-Yun Wang[a,c]*, Chengsen Cui[b]*

[a]Key Laboratory of Marine Drugs, The Ministry of Education of China, Institute of Evolution & Marine Biodiversity, School of Medicine and Pharmacy, Ocean University of China, Qingdao 266003, P. R. China

[b]Tianjin Institute of Industrial Biotechnology, Chinese Academy of Sciences, Tianjin 300308, P. R. China

[c]Laboratory for Marine Drugs and Bioproducts, Qingdao National Laboratory for Marine Science and Technology, Qingdao 266003, P. R. China

[d]State Key Laboratory of Mycology, Institute of Microbiology, Chinese Academy of Sciences, Beijing 100101, P.R. China

[e]Beijing Institute for Drug Control, NMPA Key Laboratory for Research and Evaluation of Generic Drugs, Beijing Key Laboratory of Analysis and Evaluation on Chinese Medicine, Beijing 102206, P. R. China

[f]Syngenta Jealott's Hill International Research Centre, Bracknell, Berkshire, RG42 6EY, United Kingdom

Email: cuichs@tib.cas.cn

Table of contents

Table S1. The plasmids of strain of overproducing CC and enzyme $EasD_{af}$ used in this study. 1
Table S2. Primers for $EasD_{af}$ used in this study...2
Figure S1. The sequence alignment for $EasD_{af}$ and classical SDRs..3
Figure S2. SDS-PAGE of purified $EasD_{af}$ and mutants in vitro assays 4
Figure S3. ^{1}H NMR spectrum of **1** in DMSO. 5
Figure S4. ^{1}H NMR spectrum of **2** in $CDCl_3$. 6
Figure S5. ^{1}H NMR spectrum of **3** in DMSO. 7
Figure S6. ^{13}C NMR spectrum of **3** in DMSO. 8
Figure S7. ^{1}H-^{1}H COSY spectrum of **3** in DMSO. 9
Figure S8. HSQC spectrum of **3** in DMSO. 10
Figure S9. HMBC spectrum of **3** in DMSO. 11

Table S1. The plasmids of strain of overproducing CC and enzyme $EasD_{af}$ used in this study.

Plasmids	Description	Vector backbone	References
pEA12	*PglaA: :easF – PgpdA: :dmaW – PamyB: :easE (A. fum) – Ptef1: :easC (A. fum)*	pYTU	(Yao et al. 2022)
pEA13	*PamyB: :easF – PgpdA: : easC (A. fum) – PglaA: :easE (A. fum)*	pYTP	(Yao et al. 2022)
pEA14	*P gpdA: : easC (A. fum) – PamyB: : dmaW – PglaA: :easE (A. fum)*	pYTR	(Yao et al. 2022)
Pet28a-$EasD._{af}$	$easD._{af}$	Pet28a	(Yu et al. 2022)

Table S2. Primers for $EasD_{af}$ used in this study.

Primer	Function	Sequence (5′ to 3′)[a]
Pet28a-EasC-F	pEasD Construction	AGTGGTGGTGGTGGTGGTGCTCGAGCTACGGCATGCAAGCACCGA
Pet28a-EasC-R	pEasD Construction	CGGCCTGGTGCCGCGCGGCAGCCATATGGCATCAGTCGAATCCCG
Y166A-F	$EasD_{af}$ mutation	TGAGGTGCCAGCCGCATACACGTCTGGCATGTGA
Y166A-R	$EasD_{af}$ mutation	GTGTATGCGGCTGGCACCTCAAAAGGAGCCTGCGC
S153A-F	$EasD_{af}$ mutation	CTCGCAATAGCGCCAACGTTGACAATACTTCTA
S153A-R	$EasD_{af}$ mutation	ACGTTGGCGCTATTGCGAGCGTTTCTCACATGCCA

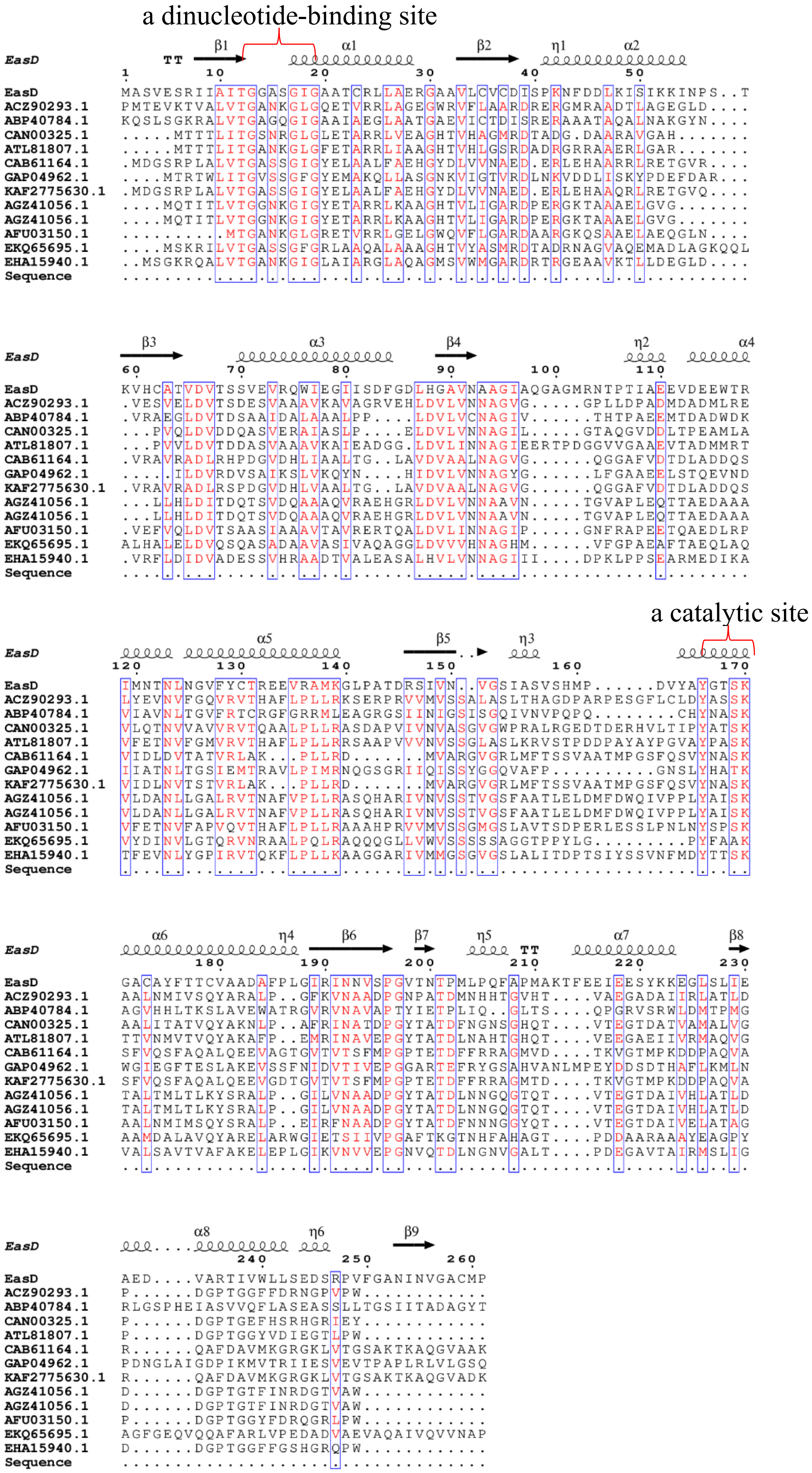

Figure S1. The sequence alignment for $EasD_{af}$ and classical SDRs.

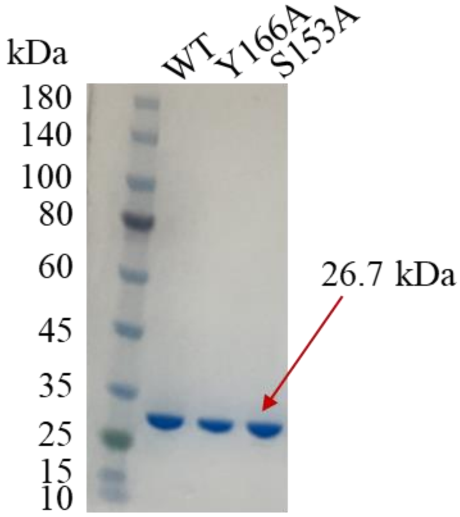

Figure S2. SDS-PAGE of purified $EasD_{af}$ and mutants in vitro assays.

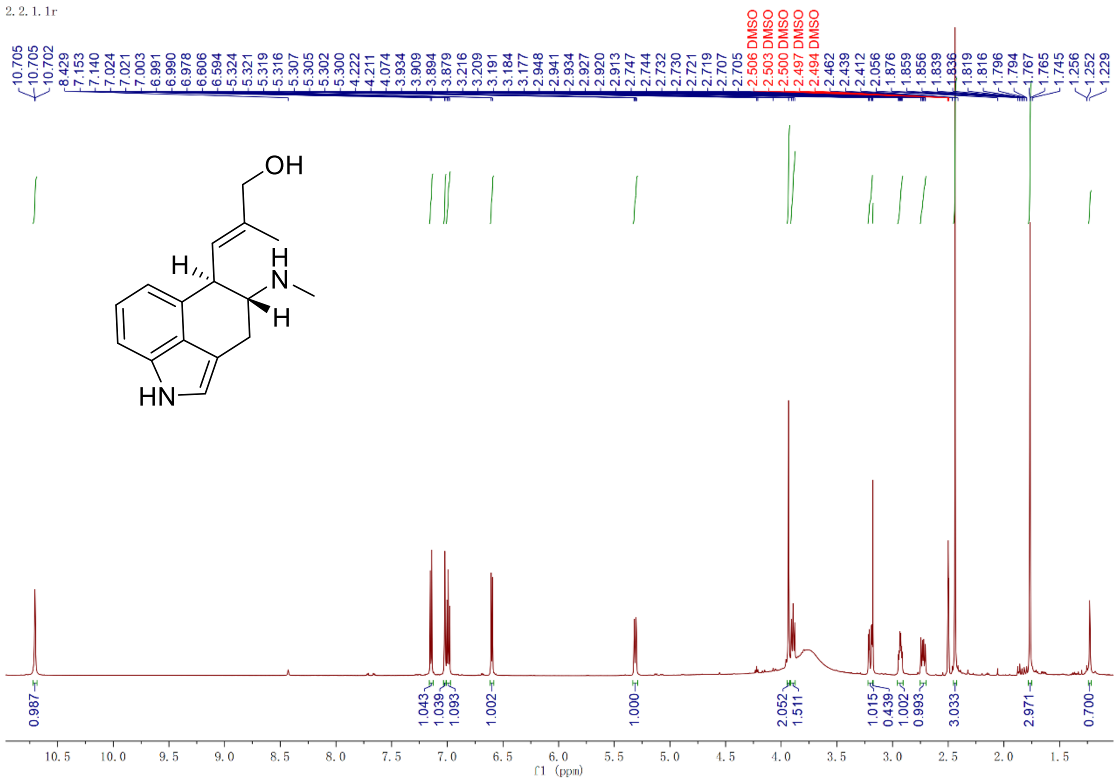

Figure S3. ^{1}H NMR spectrum of **1** in DMSO.

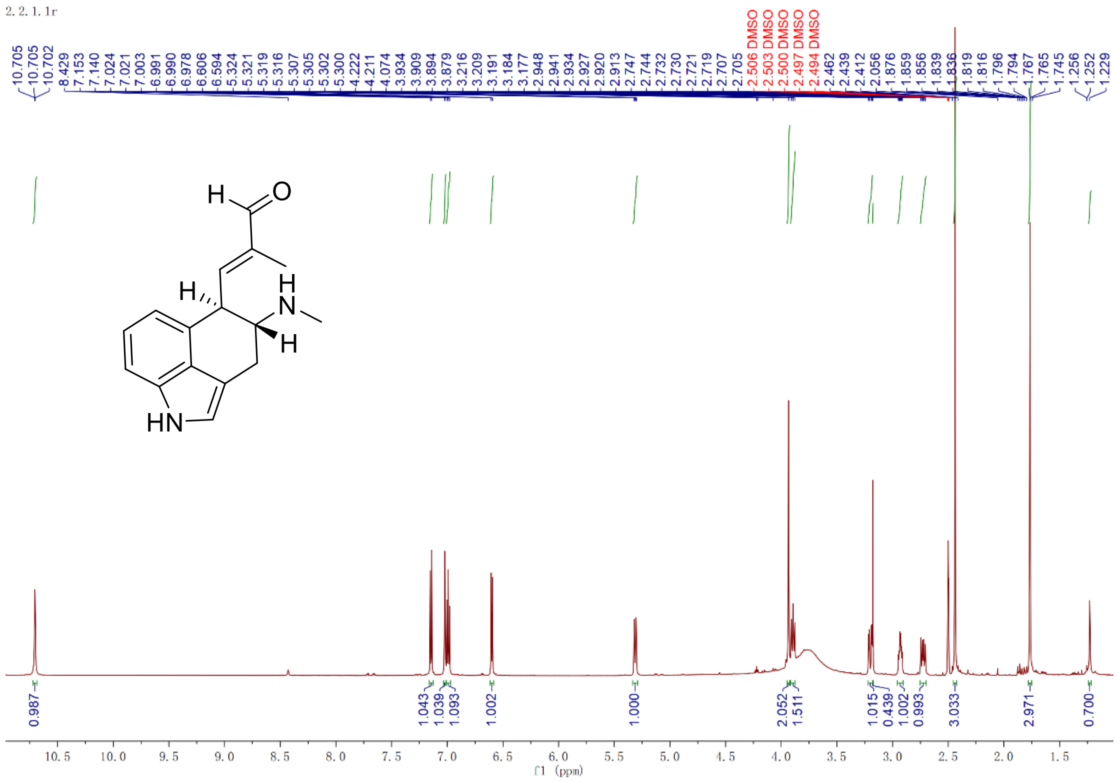

Figure S4. ^{1}H NMR spectrum of **2** in $CDCl_3$.

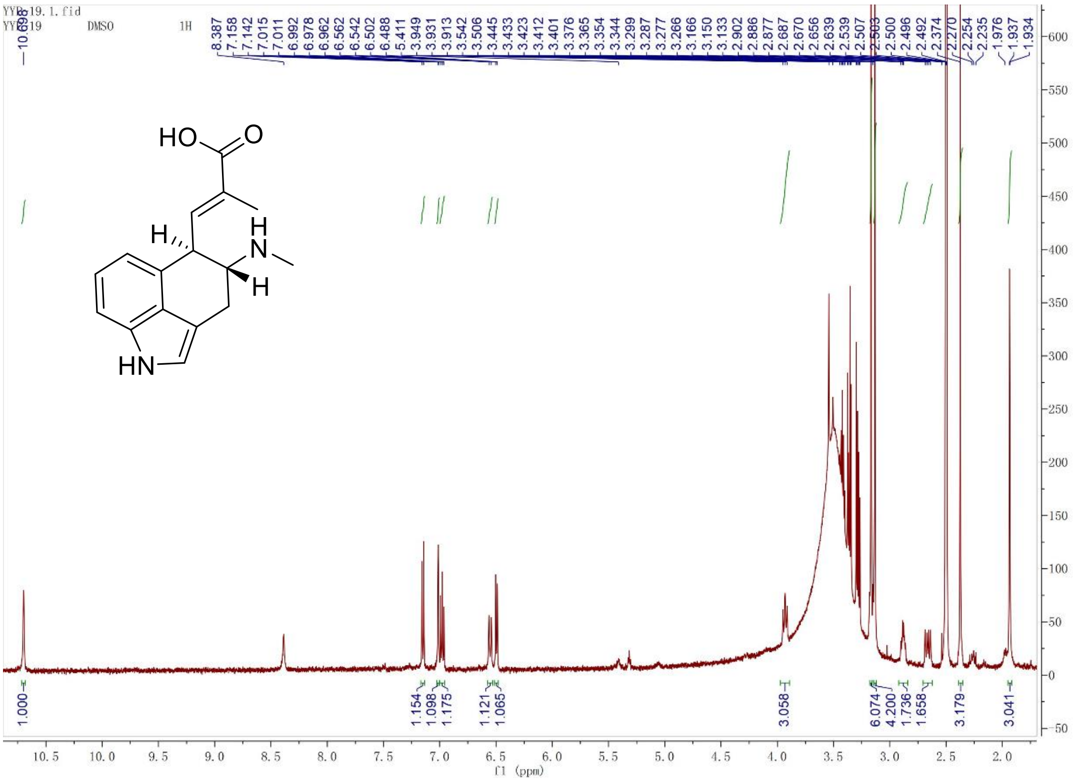

Figure S5. ^{1}H NMR spectrum of **3** in DMSO.

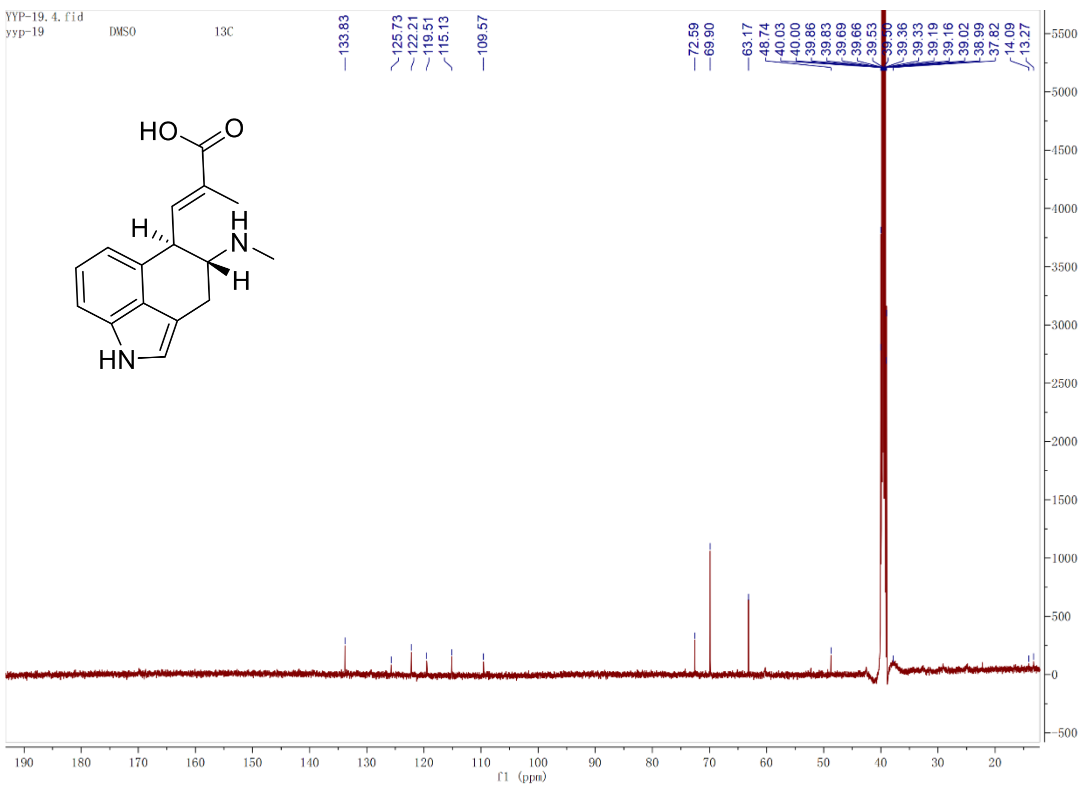

Figure S6. ^{13}C NMR spectrum of **3** in DMSO.

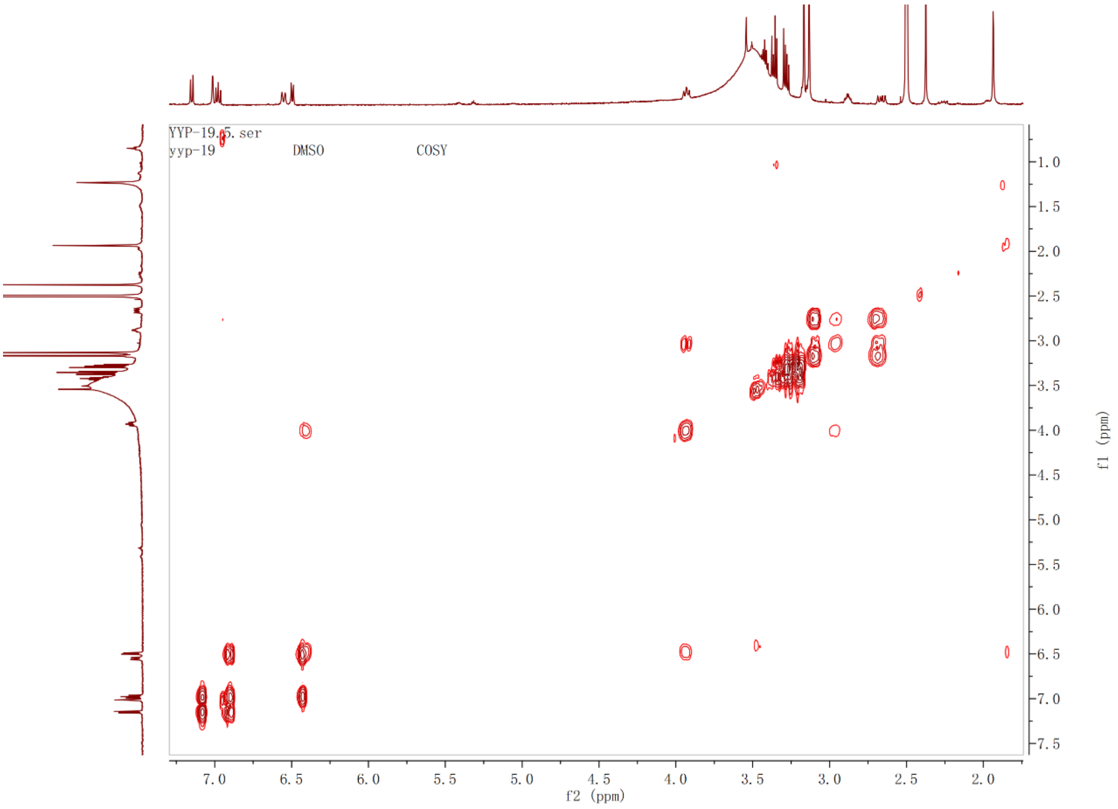

Figure S7. ^{1}H-^{1}H COSY spectrum of **3** in DMSO.

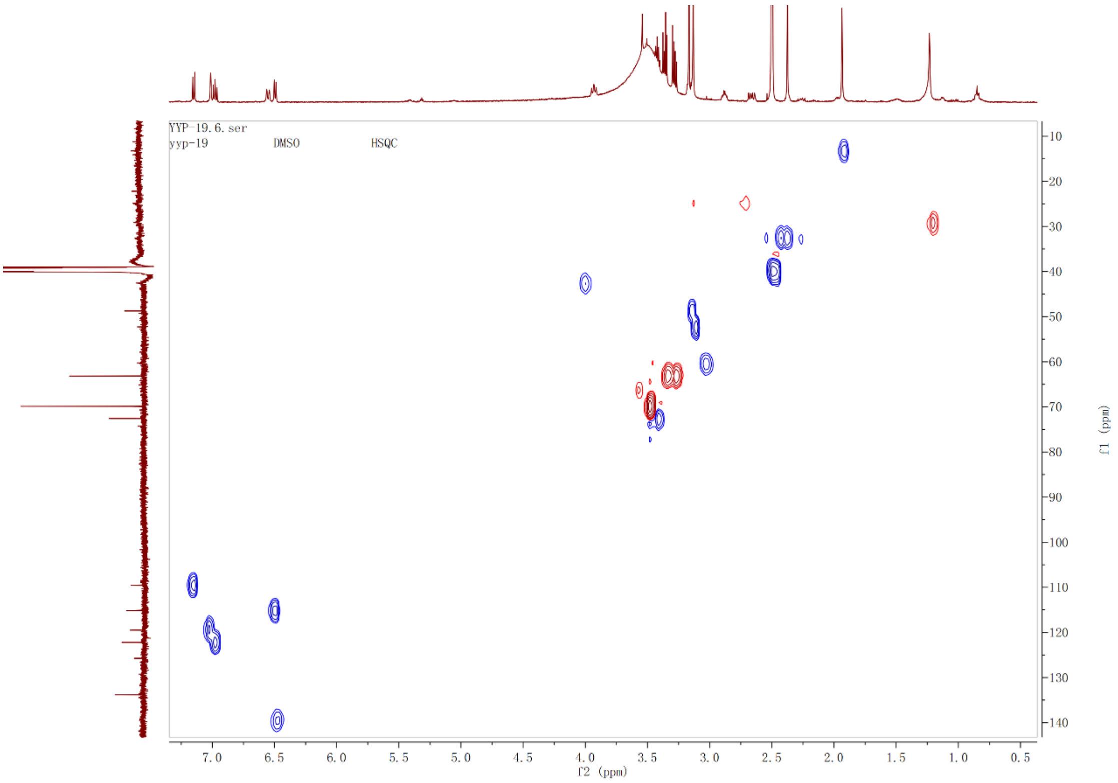

Figure S8. HSQC spectrum of **3** in DMSO.

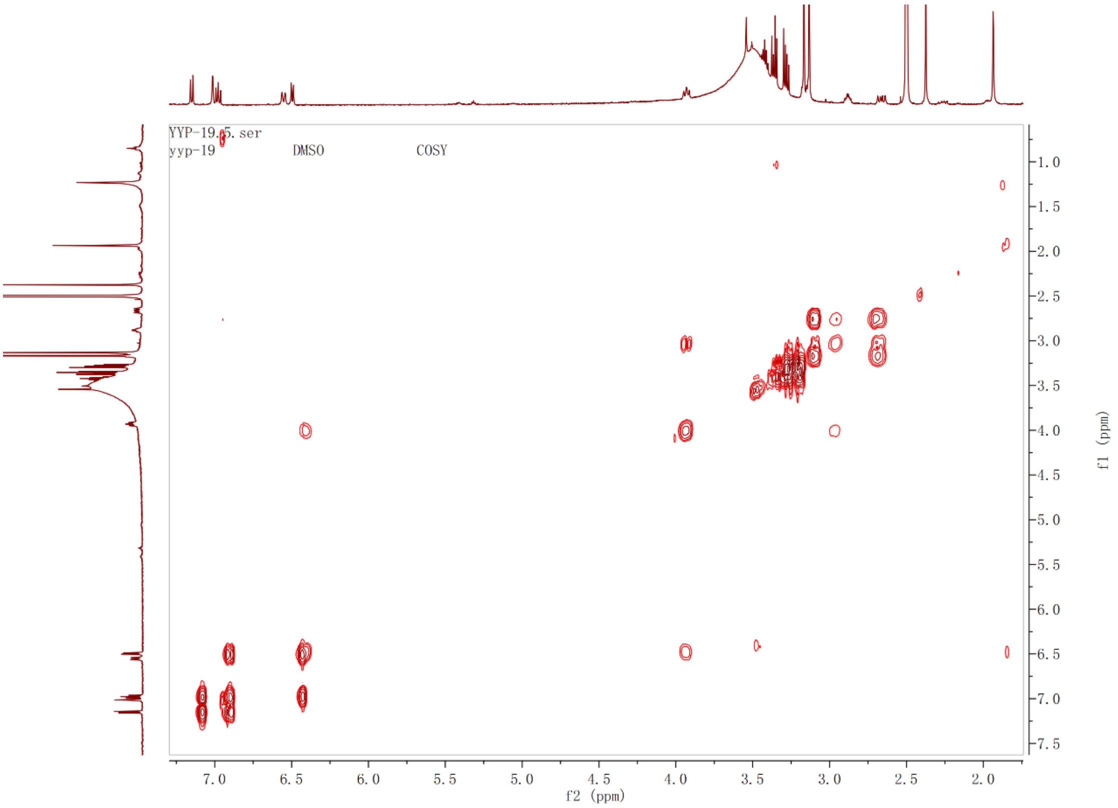

Figure S9. HMBC spectrum of **3** in DMSO.

Supplementary Reference

Yao Y, Wang W, Shi W, Yan R, Zhang J, Wei G, Liu L, Che Y, An C, Gao SS (2022) Overproduction of medicinal ergot alkaloids based on a fungal platform. Metab Eng 69:198-208. http://doi.org/https://doi.org/10.1016/j.ymben.2021.12.002.

Yu ZP, An C, Yao Y, Wang CY, Sun Z, Cui C, Liu L, Gao SS (2022) A combined strategy for the overproduction of complex ergot alkaloid agroclavine. Synth Syst Biotechnol 7(4):1126-1132. http://doi.org/10.1016/j.synbio.2022.08.003.
